# Gut microbiota in children with split-dose bowel preparations revealed by metagenomics

**DOI:** 10.3389/fcimb.2023.1202007

**Published:** 2023-07-18

**Authors:** Yu Zou, Sihui Zeng, Moxian Chen, Sufang Li, Qin Fu, Shaoming Zhou, Jianli Zhou

**Affiliations:** ^1^ Division of Gastroenterology, Shenzhen Children’s Hospital, Shenzhen, Guangdong, China; ^2^ Co-Innovation Center for Sustainable Forestry in Southern China & Key Laboratory of National Forestry and Grassland Administration on Subtropical Forest Biodiversity Conservation, College of Biology and the Environment, Nanjing Forestry University, Nanjing, China; ^3^ Nursing Department, Shenzhen Children’s Hospital, Shenzhen, Guangdong, China

**Keywords:** bowel preparation, children, colonoscopy, gut microbiota, metagenomics, resilience, split dose, stability

## Abstract

**Objective:**

Split-dose polyethylene glycol (PEG) is routinely used for bowel preparation before colonoscopy. This study aimed to investigate the composition of gut microbiota and its functions in pediatric patients undergoing split-dose PEG bowel preparation for colonoscopy to understand the stability and resilience of gut microbiota.

**Material and methods:**

From September to December 2021, 19 pediatric patients were enrolled at Shenzhen Children’s Hospital and 76 samples (4 time points) were analyzed using metagenomics. Time points included Time_1 (one day before bowel preparation), Time_2 (one day after colonoscopy), Time_3 (two weeks after bowel preparation), and Time_4 (four weeks after bowel preparation).

**Result:**

Alpha diversity comparison at both the species and gene levels showed a decrease in community richness after colonoscopy, with little statistical significance. However, the Shannon diversity index significantly decreased (*P*<0.05) and gradually returned to pre-preparation levels at two weeks after bowel preparation. The genus level analysis showed six genera (*Eubacterium*, *Escherichia*, *Intertinibacter*, *Veillonella*, *Ruminococcaceae unclassified*, and *Coprobacillus*) significantly different across the four time periods. Additionally, at the species level, the abundance of *Escherichia coli*, *Bacteroides fragilis*, and *Veillonella parvula* significantly increased at one day after colonoscopy before gradually decreasing at two weeks after bowel preparation. In contrast, the abundance of *Intertinibacter bartlettii* decreased at one day after colonoscopy but then recovered at two weeks after bowel preparation, reaching the preoperative level at four weeks after bowel preparation. Furthermore, five functional pathways (base excision repair, biosynthesis of ansamycins, biosynthesis of siderophore group nonribosomal peptide, flavonoid biosynthesis, and biosynthesis of type II polyketide products) were significantly different across the four time periods, with recovery at two weeks after bowel preparation and reaching preoperative levels at four weeks after bowel preparation.

**Conclusions:**

Gut microbiota at the genus level, species level, and functional pathways are impacted in pediatric patients undergoing split-dose PEG bowel preparation and colonoscopy, with recovery two weeks following bowel preparation. However, the phylum level was not impacted. Modifications in gut microbiota composition and function may be investigated in future studies of bowel preparation. This study highlights the stability and resilience of gut microbiota among pediatric patients during bowel preparation.

## 1 Introduction

Colonoscopy is a commonly used method for screening and treating intestinal diseases in children. Achieving good preparation of the bowel is crucial for improving diagnostic and therapeutic outcomes. The drug of choice for bowel preparation is polyethylene glycol (PEG) due to its high efficacy, safety, and ease of use, as confirmed by several studies. Both adult and child guidelines recommend PEG as the preferred laxative for bowel preparation (Pashankar et al., 2004; Safder et al., 2008; Pall et al., 2014; ASGE Standards of Practice Committee et al., 2015; Tsunoda et al., 2017; Mytyk et al., 2018). Split-dose PEG is commonly used for bowel preparation in adults before colonoscopy (Samarasena et al., 2012; Zawaly et al., 2019). Research has shown that split-dose PEG is more effective than a single dose for bowel preparation in children (Tripathi et al., 2020).

The human microbiome is composed of various microorganisms, including bacteria, archaea, fungi, protozoa, and viruses, with more than 100 times the number of genes as the human body (Qin et al., 2010; Ursell et al., 2012). These genes encode pathways that produce biologically active molecules from diet and metabolism (Lynch and Pedersen, 2016). The gut microbiota plays a vital role in obtaining energy from dietary fiber and linking its metabolites to the occurrence of various diseases, such as irritable bowel syndrome (IBS) and inflammatory bowel disease (IBD) (Shreiner et al., 2015). Although previous studies have shown that colonoscopy and bowel preparation affect gut microbiota, there is still limited research in children (Drago et al., 2019). Furthermore, the impact of split-dose PEG bowel preparation on changing gut microbiota in children has not been well studied, and there are no metagenomic studies in this area. To obtain a more comprehensive view of the microbial community, metagenomics was employed for investigating gut microbiota of children in this study. Metagenomics can detect both bacterial and non-bacterial microorganisms, providing information about the functional potential of the microbial community. In contrast, 16S rRNA sequencing only detects bacterial microorganisms and does not provide functional potential information. Thus, our study aimed to characterize the potential differences in gut microbiota composition and functional capacity in children undergoing colonoscopy with split-dose PEG bowel preparation.

## 2 Results

### 2.1 Alpha diversity analysis between the 4 time points at the species and gene levels

**Figure 1:**
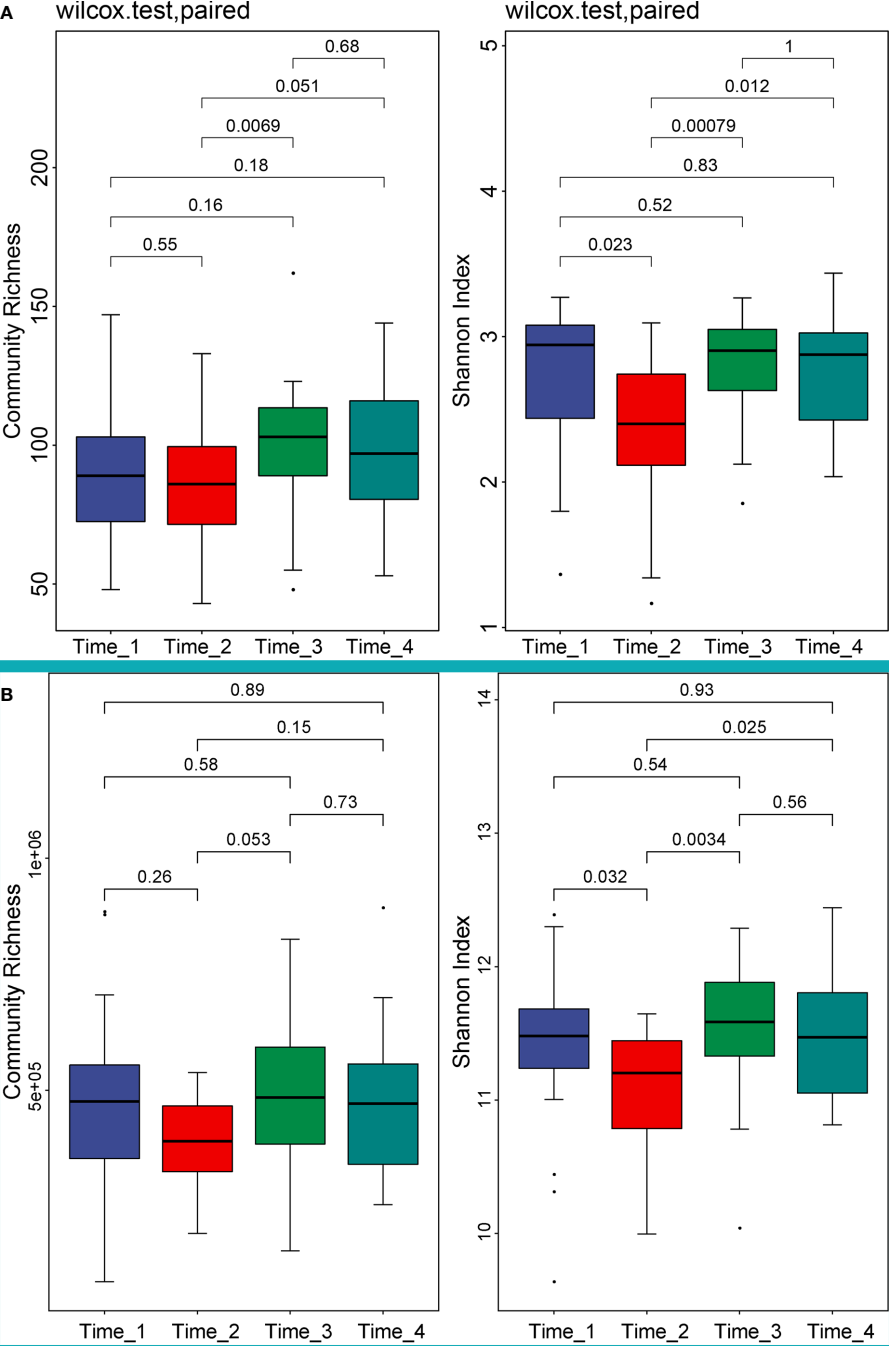
Alpha diversity analysis of 4 time points at the species and gene levels. (Left of **A**) Community richness at the species level showed that the abundance declined after bowel preparation, but not significantly. (Right of **A**) The Shannon diversity index decreased significantly at the species level (*P*=0.023) and gradually returned to the level before bowel preparation at Time_3. (Left of **B**) Community richness at the gene level showed that the abundance declined after bowel preparation, but not significantly. (Right of **B**) The Shannon diversity index decreased significantly at the gene level (*P*=0.032) and gradually returned to the level before bowel preparation at Time_3. Note, The Wilcoxon rank sum test was used to test the difference between groups, and the value above the horizontal line was the P-value of the difference test. Time_1: one day before bowel preparation; Time_2: two days after bowel preparation (one day after colonoscopy); Time_3: two weeks after bowel preparation; Time_4: four weeks after bowel preparation.

We compared alpha diversity at the species and gene levels between the 4 time points, as detailed in **Table S2**. Our analysis showed that although abundance decreased after bowel preparation, it was not significant (**Figures 1A, B**). However, the Shannon diversity index significantly decreased (*P*<0.05) at both the species and gene levels, which gradually returned to baseline levels by two weeks after bowel preparation (**Figures 1A, B**).

### 2.2 Beta diversity analysis between the 4 time points

**Figure 2:**
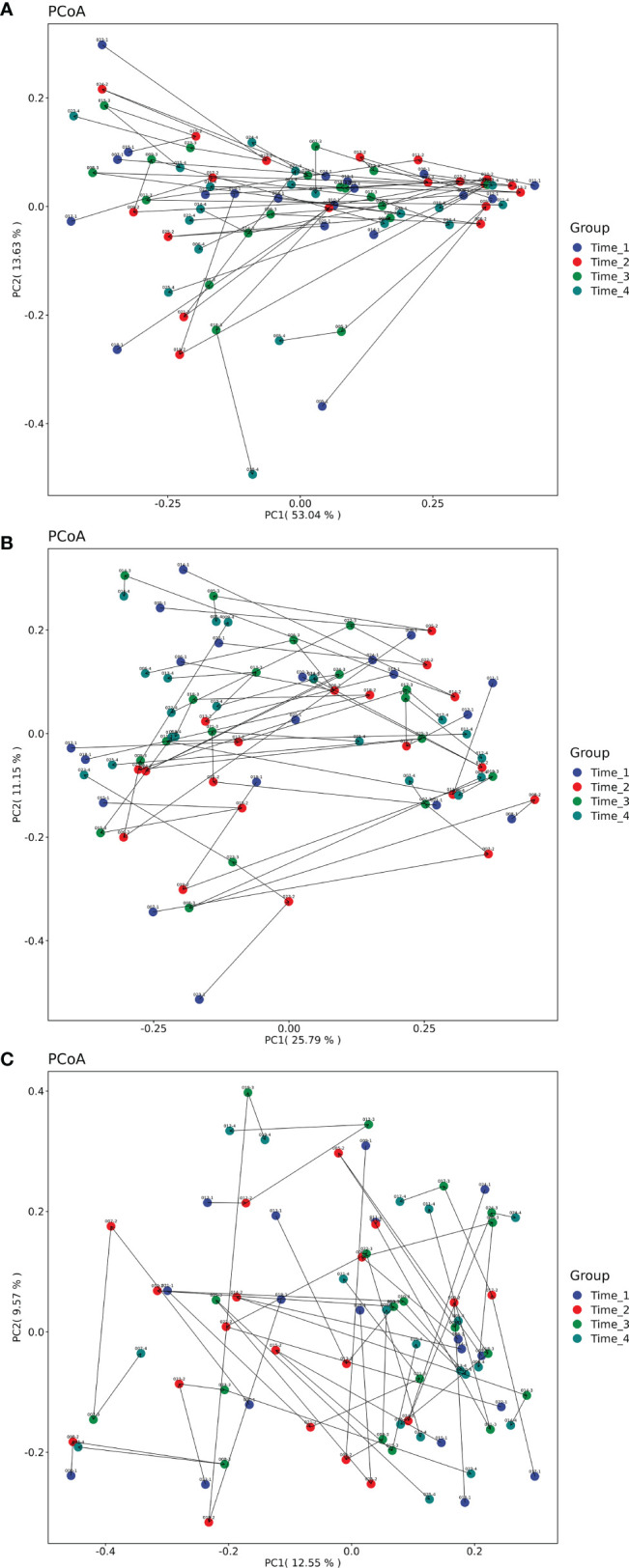
PCoA analysis based on Bray-Curtis distance of microbiota composition at 4 time points. **(A)** Beta diversity analysis of 4 time points at the phylum level. There was no significant difference in beta diversity between the 4 time points at the phylum level (*P*=0.564). **(B)** Beta diversity analysis of 4 time points at the genus level. There was no significant difference in beta diversity between the 4 time points at the genus level (*P*=0.545). **(C)** Beta diversity analysis of 4 time points at the species level. There was no significant difference in beta diversity between the 4 time points at the species level (*P*=0.624). None of the dots overlapped. Note, Different colored dots indicate different times. The line and arrow are connected for the same patient with sample ID (detailed in **Table S1**). Time_1: one day before bowel preparation; Time_2: two days after bowel preparation (one day after colonoscopy); Time_3: two weeks after bowel preparation; Time_4: four weeks after bowel preparation.

There was no significant difference in beta diversity between the 4 time points at the phylum level (*P*=0.564), genus level (*P*=0.545) and species level (*P*=0.624) (**Figure 2**). Moreover, PCoA based on Bray-Curtis distance was used to compare the changes in microbiota composition at different time points. The dots did not overlap, suggesting that the composition of the microbiota was altered after bowel preparation.

### 2.3 Analysis of differential microbiota at the phylum level

**Figure 3:**
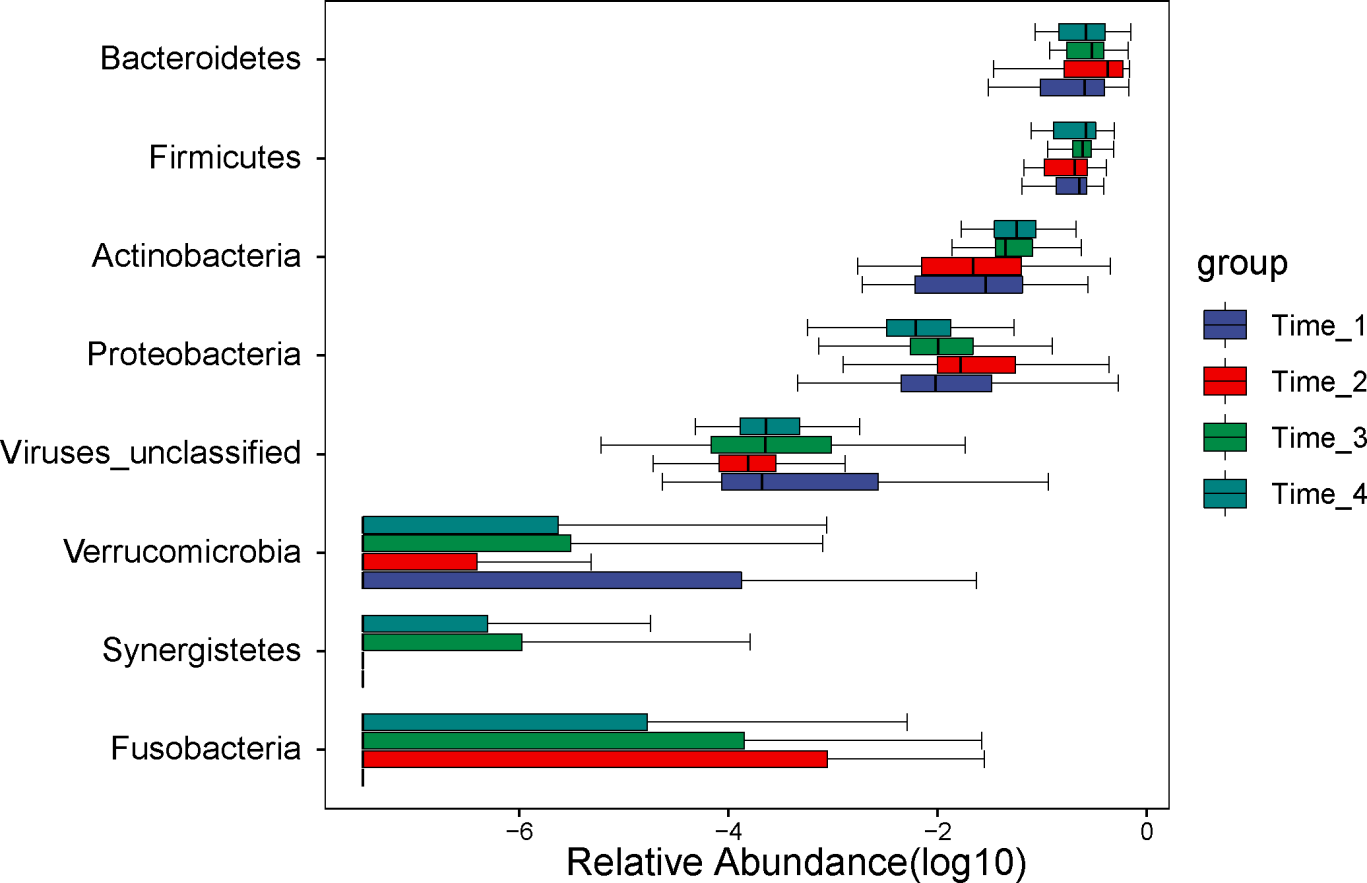
Analysis of differential microbiota at the phylum level. No differential microbiota was found at the phylum level (*P*>0.05). Note, Time_1: one day before bowel preparation; Time_2: two days after bowel preparation (one day after colonoscopy); Time_3: two weeks after bowel preparation; Time_4: four weeks after bowel preparation.

A comparison of gut microbial compositional features at the phylum level between the 4 time points (detailed data in **Table S3**). No significant difference was observed at the phylum level among the four time points (*P*>0.05) (**Figure 3**).

### 2.4 Analysis of differential microbiota at the genus level

**Figure 4:**
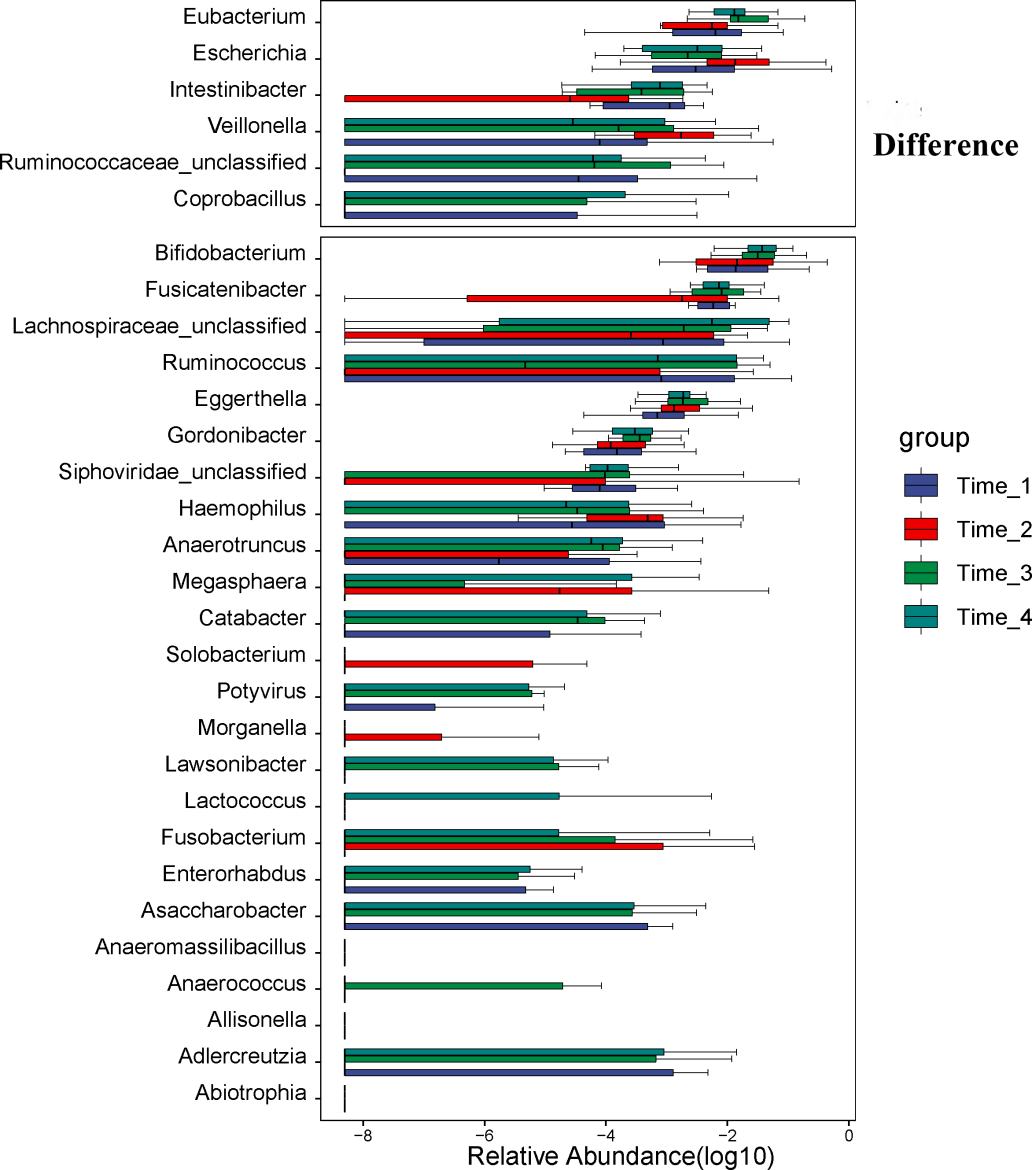
Analysis of differential microbiota at the genus level. At the genus level, six genera (*Eubacterium*, *Escherichia*, *Intertinibacter*, *Veillonella*, *Ruminococcaceae_unclassified* and *Coprobacillus*) were significantly different between the 4 time points. The abundance of *Eubacterium* increased at Time_3. While the abundance of *Escherichia* and *Veillonella* increased at Time_2 and then gradually returned to the level before bowel preparation at Time_3, the abundance of *Intertinibacter* decreased at Time_2 and was restored to the level before bowel preparation at Time_3 and Time_4. Note: Time_1: one day before bowel preparation; Time_2: two days after bowel preparation (one day after colonoscopy); Time_3: two weeks after bowel preparation; Time_4: four weeks after bowel preparation.

A comparison of gut microbial compositional features at the genus level between the 4 time points (detailed data in **Table S4**). At the genus level, six genera (*Eubacterium*, *Escherichia*, *Intertinibacter*, *Veillonella*, *Ruminococcaceae_unclassified* and *Coprobacillus*) were significantly different between the 4 time points (**Figure 4**). The abundance of *Eubacterium* increased at two weeks after bowel preparation. While the abundance of *Escherichia* and *Veillonella* increased at two days after bowel preparation and then gradually returned to the baseline level at two weeks after bowel preparation, the abundance of *Intertinibacter* decreased at two days after bowel preparation and was restored to the baseline level at two weeks and four weeks after bowel preparation.

### 2.5 Analysis of differential microbiota at the species level

**Figure 5:**
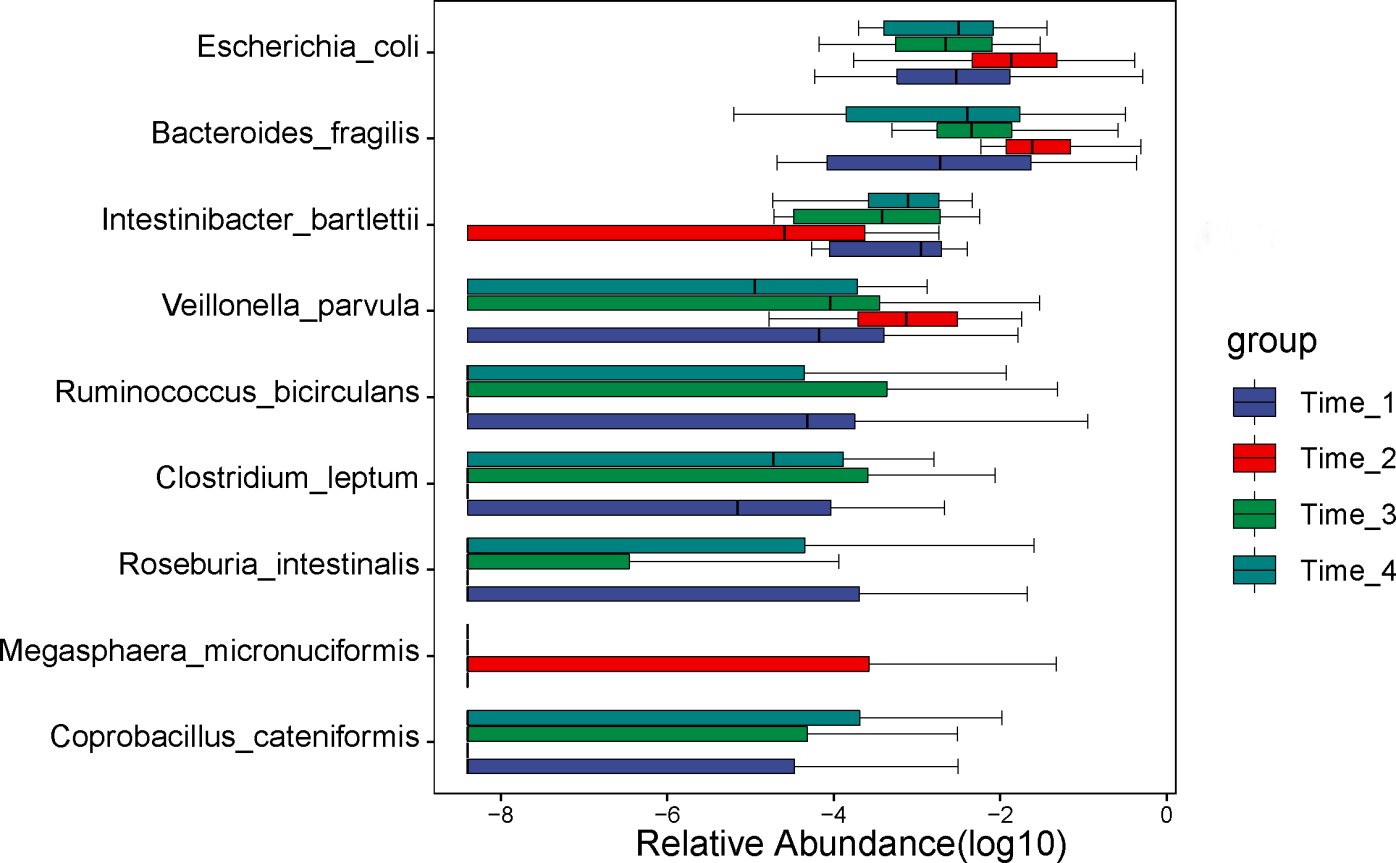
Analysis of differential microbiota at the species level. At the species level, we discovered that the abundance of *Escherichia coli*, *Bacteroides fragilis* and *Veillonella parvula* increased significantly at Time_2 and gradually decreased at Time_3. The abundance of *Intertinibacter bartlettii* decreased obviously at Time_2, recovered at Time_3, and approached the level before bowel preparation at Time_4. Note: Time_1: one day before bowel preparation; Time_2: two days after bowel preparation (one day after colonoscopy); Time_3: two weeks after bowel preparation; Time_4: four weeks after bowel preparation.

A comparison of gut microbial compositional features at the species level between the 4 time points (detailed data in **Table S5**). At the species level, we discovered that the abundance of *Escherichia coli* (*P*=0.028), *Bacteroides fragilis* (*P*=0.039) and *Veillonella parvula* (*P*=0.002) increased significantly at two days after bowel preparation and gradually decreased at two weeks after bowel preparation (**Figure 5**). The abundance of *Intertinibacter bartlettii* (*P*=0.003) decreased obviously at two days after bowel preparation, recovered at two weeks after bowel preparation, and approached the baseline level at four weeks after bowel preparation.

### 2.6 KEGG functional pathway analysis

**Figure 6:**
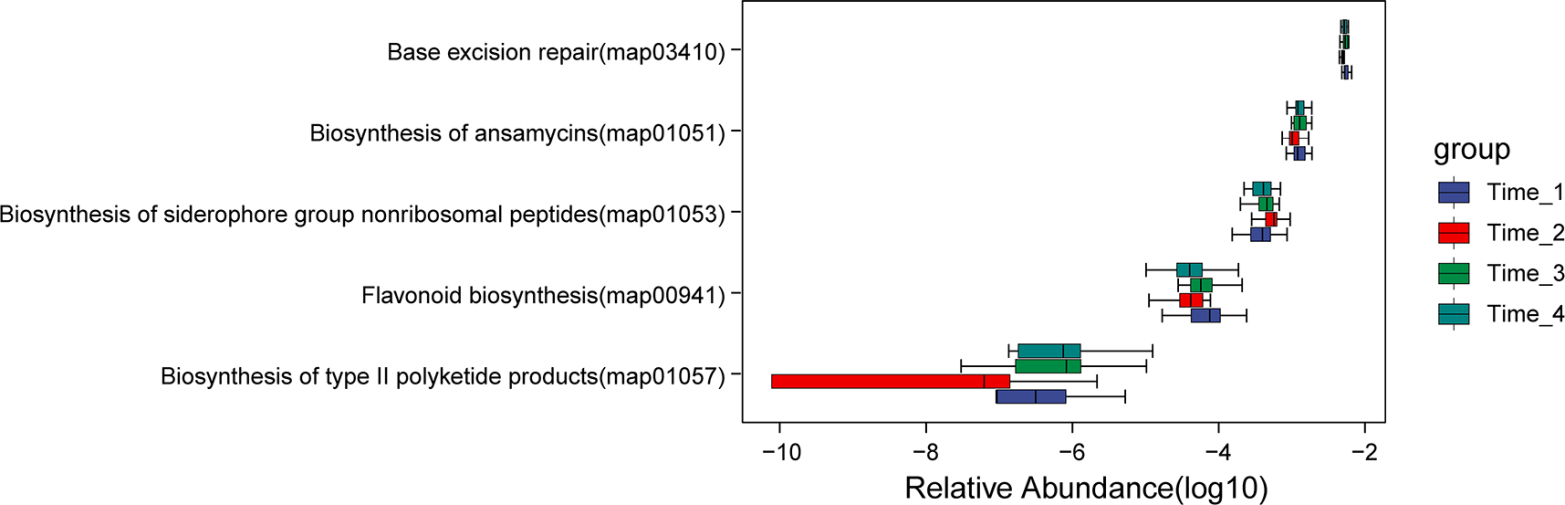
Analysis of differential functional pathways. Five functional pathways (base excision repair, biosynthesis of ansamycins, biosynthesis of siderophore group nonribosomal peptide, flavonoid biosynthesis and biosynthesis of type II polyketide products) were significantly different in the four time periods (*P*<0.05). While the pathway of biosynthesis of siderophore group nonribosomal peptide was decreased obviously at Time_2, other pathways were increased significantly at Time_2, but all of them recovered at Time_3 and approached the level before bowel preparation at Time_4. Note: Time_1: one day before bowel preparation; Time_2: two days after bowel preparation (one day after colonoscopy); Time_3: two weeks after bowel preparation; Time_4: four weeks after bowel preparation.

The Kruskal-Wallis test was used, and *P*<0.05 was used as the screening condition for significant differences in functional pathways (detailed data in **Table S6**). Five functional pathways (base excision repair, biosynthesis of ansamycins, biosynthesis of siderophore group nonribosomal peptide, flavonoid biosynthesis and biosynthesis of type II polyketide products) were significantly different in the four time periods (*P*<0.05) (**Figure 6**). While the pathway of biosynthesis of siderophore group nonribosomal peptide was decreased obviously at two days after bowel preparation, other pathways were increased significantly at two days after bowel preparation, but all of them recovered at two weeks after bowel preparation and approached the baseline level at four weeks after bowel preparation.

## 3 Discussion

The impact of bowel preparation on the composition of gut microbiota was first reported by Mai’s team in 2006 (Mai et al., 2006). Since then, there have been a total of studies on this subject, with only one study reported in children (**Table 1**) (Mai et al., 2006; Harrell et al., 2012; Gorkiewicz et al., 2013; O'Brien et al., 2013; Jalanka et al., 2015; Drago et al., 2016; Shobar et al., 2016; Shaw et al., 2017; Powles et al., 2022). These studies comprised a sample size ranging from 4 to 23 and included both healthy and those with diseases such as IBD. Most of the studies utilized 16S rRNA, with no reported use of metagenomics. Thus, our study was the first to utilize metagenomics in investigating the effects of split-dose PEG bowel preparation and colonoscopy on gut microbiota in children.

**Table 1:** Research associated with the effects of bowel preparation on gut microbiota.

Research (ref)	Age (years)	Mean BMI (kg/cm^2^)	No. patients	Bowel cleansing	Sample	Sampling time	Detection method	Result
Present research	10.01 ± 3.47	17.36	19 children	Split-dose PEG	Stool	Before bowel preparation after two days after two weeks after four weeks	Metagenomics	The genus level, species level and functional pathways were affected, but they recovered two weeks later. The microbiota did not change at the phylum level.
Mai et al. (21)	/	/	5 adults	/	Stool	Before colonoscopy during Colonoscopy after 6-8 weeks	DGGE	The composition of microbiota was disturbed in patients undergoing colonoscopy.
Harrell et al. (22)	25-48	/	12 healthy adults	PEG 4 L	Mucosa	Before colonoscopy after 1 week	16S rRNA sequencing	The phylum level was not significantly changed, but the genus level was differences observed.
O’Brien et al. (23)	46-69	/	20 adults	PEG 2 L + bisacodyl 10 mg	Stool	Before colonoscopy after 1 week after 4 weeks after 12-24 weeks	DGGE and 16S rRNA sequencing	Bowel preparation did not have a lasting influence on the composition of the microbiota.
Gorkiewicz et al. (24)	36-47	24-26.6	4 healthy adults	PEG 150 g, for 3 days	Stool and mucosa	Before colonoscopy after 4 days	16S rRNA sequencing	The phylum level was significantly changed both in the stool and mucosa.
Jalanka et al. (25)	25-27	23-23.3	23 healthy adults	PEG 1 L × 2 vs 2 L	Stool	Before colonoscopy during colonoscopy after 2 weeks after 4 weeks	16S rRNA sequencing	The split-dose bowel preparation introduced fewer effects to the gut microbiota than a single dose. The composition of the microbiota was decreased, and they would restored within 14 days, the rate of recovery was dose dependent.
Drago et al. (26)	40-68	24.6	10 adults	PEG 4 L	Stool	Before colonoscopy during colonoscopy after 4 weeks	16S rRNA sequencing	The gut microbiota at the phylum, class, and family level were changed.
Shobar et al. (27)	49-55.4	22.15-32.2	8 IBD and 10 healthy adults	/	Stool and mucosa	Before colonoscopy during colonoscopy	16S rRNA sequencing	The composition and diversity of the fecal and luminal microbiota were affected.
Shaw et al. (28)	4-17	/	16 children	Sodium picosulfate with magnesium citrate and senna	Stool, mucosa, swab	Before colonoscopy during colonoscopy after 54 days	16S rRNA sequencing	Bowel preparation had a clear transient effect on the microbiota during colonoscopy, but no significant long-term effect.
Powles et al. (29)	41	23.4	11 adults	MoviPrep	Stool and urine	Before colonoscopy after 3 days after 6 weeks	16S rRNA sequencing	Bowel preparation temporarily reduced the alpha diversity of gut microbiota without significant changes in fecal and urine metabolites.

PEG, polyethylene glycol; DGGE, denaturing gradient gel electrophoresis;/, not mention; IBD, inflammatory bowel disease; MoviPrep, included Macrogol 3350, sodium sulfate anhydrous, sodium chloride, potassium chloride, ascorbic acid and sodium ascorbate; our study is highlighted in red.

Our study revealed a decrease in alpha diversity on the second day after bowel preparation, which gradually returned to the level before bowel preparation over the following two weeks. This finding is consistent with another study that reported a decrease in alpha diversity three days after bowel preparation that returned to pre-preparation levels at six weeks (Powles et al., 2022). Additionally, another study reported a reduction in the Shannon index only in biopsy samples from IBD patients (Shobar et al., 2016). These results suggest that bowel preparation does indeed affect alpha diversity, but its levels return to normal over time. However, beta diversity was not influenced by bowel preparation at the phylum, genus, and species levels. We observed distinct dots, which indicates that the composition of gut microbiota was altered after bowel preparation. To date, no studies have reported on the effect of bowel preparation and colonoscopy on beta diversity of gut microbiota.

Our study did not reveal any significant difference at the phylum level, which was consistent with previous research (Harrell et al., 2012). However, some studies have reported changes at the phylum level following bowel preparation (Gorkiewicz et al., 2013; Jalanka et al., 2015; Drago et al., 2016; Shaw et al., 2017). Notably, in these studies, the PEG dose was relatively high or a single dose was used. It should be noted that one of these studies reported that bowel preparation using a single dose led to more alterations in gut microbiota than two separate doses (Jalanka et al., 2015). This suggests that split-dose PEG bowel preparation only has a minor effect on gut microbiota at the phylum level.

At the genus level, we found that six genera, including *Eubacterium*, *Escherichia*, *Intertinibacter*, *Veillonella*, *Ruminococcaceae_unclassified* and *Coprobacillus*, were significantly different. Some increased after bowel preparation, while some decreased, but most of them returned to normal after 2 weeks. Many studies have reported changes at the genus level after bowel preparation (Harrell et al., 2012; Jalanka et al., 2015; Shobar et al., 2016; Shaw et al., 2017). Each study reported that different genera are changed after bowel preparation. A pediatric study showed a significant increase in *Faecalibacterium* and significant decreases in *Ruminococcus*, *Escherichia*, *Pseudobutyrivibrio* and *Subdoligranulum* (Shaw et al., 2017). This was different from what we reported. This may be related to the origin of the patients. Although our two subjects were about the same age, we took more stool samples than that study. We collected 19 stool samples at each time period, but that study collected 11 pre-bowel preparation stool samples, 7 post-bowel preparation stool samples, seventeen biopsy samples, sixteen luminal content samples and eighteen swabs taken at colonoscopy.

At the species level, we discovered that the abundance of *Escherichia coli*, *Bacteroides fragilis* and *Veillonella parvula* increased significantly at two days after bowel preparation, gradually decreased two weeks later and gradually recovered. The abundance of *Intertinibacter bartlettii* decreased obviously at two days after bowel preparation, recovered at two weeks later, and approached the preoperative level at four weeks. To date, no reports have been published on the effects of bowel preparation and colonoscopy on gut microbiota at the species level.

In our final analysis, we evaluated the functional diversity of gut microbiota at time points during bowel preparation. Our findings revealed a distinct decrease in the pathway of biosynthesis of siderophore group nonribosomal peptide two days after bowel preparation. Additionally, four pathways (baseision repair, biosynthesis of ansamycins, flavonoid biosynthesis, and biosynthesis of type II polyketide products) increased two days after bowel preparation, but all pathways recovered their pre-preparation levels within two weeks and gradually continued to recover. Our findings indicate that bowel preparation can decrease the biosynthesis of siderophore group nonribosomal peptide, while enhancing base excision repair, biosynthesis of ansamycins, flavonoid biosynthesis, and biosynthesis of type II polyketide products. Such effects of bowel preparation on gut microbiota have not been reported previously.

The results of our study show that after bowel preparation, the composition and functional pathways of gut microbiota undergo a change, which then gradually returns to baseline, demonstrating the stability and resilience of gut microbiota. Such qualities are considered basic ecological characteristics of gut microbiota (Lozupone et al., 2012). The ability of gut microbiota to restore its balance after infection with a pathogen or antibiotic treatment is referred to as resilience (Sommer et al., 2017; Palleja et al., 2018). The gut microbiota’s stability and resilience are affected by factors such as unhealthy status, antibiotic use, and dietary factors (Fassarella et al., 2021). Our study represents the first utilization of metagenomics in children with split-dose PEG bowel preparation to understand the stability and resilience of gut microbiota. We also discovered that the primary outcome across studies in this field both in children and adults is the compositional alteration of gut microbiota after bowel preparation and colonoscopy, after which it recovers with time. Our findings are generally consistent with previous studies, indicating that split-dose PEG can be widely utilized for bowel preparation in children.

Our study had two limitations. Firstly, no detailed food frequency questionnaires were included which could account for the potential influence of diet on the observed differences in gut microbiota. Secondly the disease spectrum of the subjects was not consistent, which may have influenced the results.

In conclusion, our research indicates that split-dose PEG bowel preparation and colonoscopy induce changes in gut microbiota of children at the genus and species levels, as well as in functional pathways. However, the phylum level remains unaffected. It is possible that future bowel preparation research may target these compositional and functional changes in gut microbiota. Our study also exhibits the stability and resilience of gut microbiota, laying the foundation for future research.

## 4 Materials and methods

### 4.1 Study subjects

This study was carried out with the approval of the Human Ethics Committee of Shenzhen Children’s Hospital. Participants aged from 2.5 to 16.8 years old (average age of 10.01 ± 3.47 years) were enrolled, including 11 boys and 8 girls, from September to December 2021 at Shenzhen Children’s Hospital (**Table 2**). Before colonoscopy, these patients received a split dose of PEG electrolyte solution (total 80 mL/kg, ≤ 3000 mL, divided into two parts, two-thirds and one-third); the two-thirds in the evening and the one-third on the following morning. The mean BMI was 17.36 kg/m2. Patients who recently (past 90 days) used probiotics or antibiotics were excluded.

**Table 2:** Characteristics of the study cohort.

Case	Age (years)	Gender (Male : Female 11:8)	Height (cm)	Weight (kg)	BMI (kg/cm^2^)
Case1	12.1	Male	156	49.5	20.34
Case2	10.1	Male	135	27.0	14.81
Case3	12.9	Male	161	59.8	23.07
Case4	12.9	Male	154	43.4	18.29
Case5	13.6	Female	153	45.1	19.26
Case6	2.5	Male	97	15.2	16.15
Case7	13.4	Female	164	46.2	17.17
Case8	9.4	Female	128	21.4	13.06
Case9	6.2	Female	123	19.4	12.82
Case10	10	Female	149	36.5	16.44
Case11	16.8	Male	175	83.6	27.29
Case12	9.0	Male	139	33.2	17.18
Case13	7.0	Female	128	23.4	14.28
Case14	7.0	Female	126	23.6	14.86
Case15	9.0	Male	142	29.2	14.48
Case16	9.0	Female	140	39.6	20.20
Case17	12.9	Male	157	57.0	23.12
Case18	5.2	Male	112	16.4	13.07
Case19	11.1	Male	131	24.0	13.98

### 4.2 Sample collection and metagenomic sequencing

**Figure 7:**
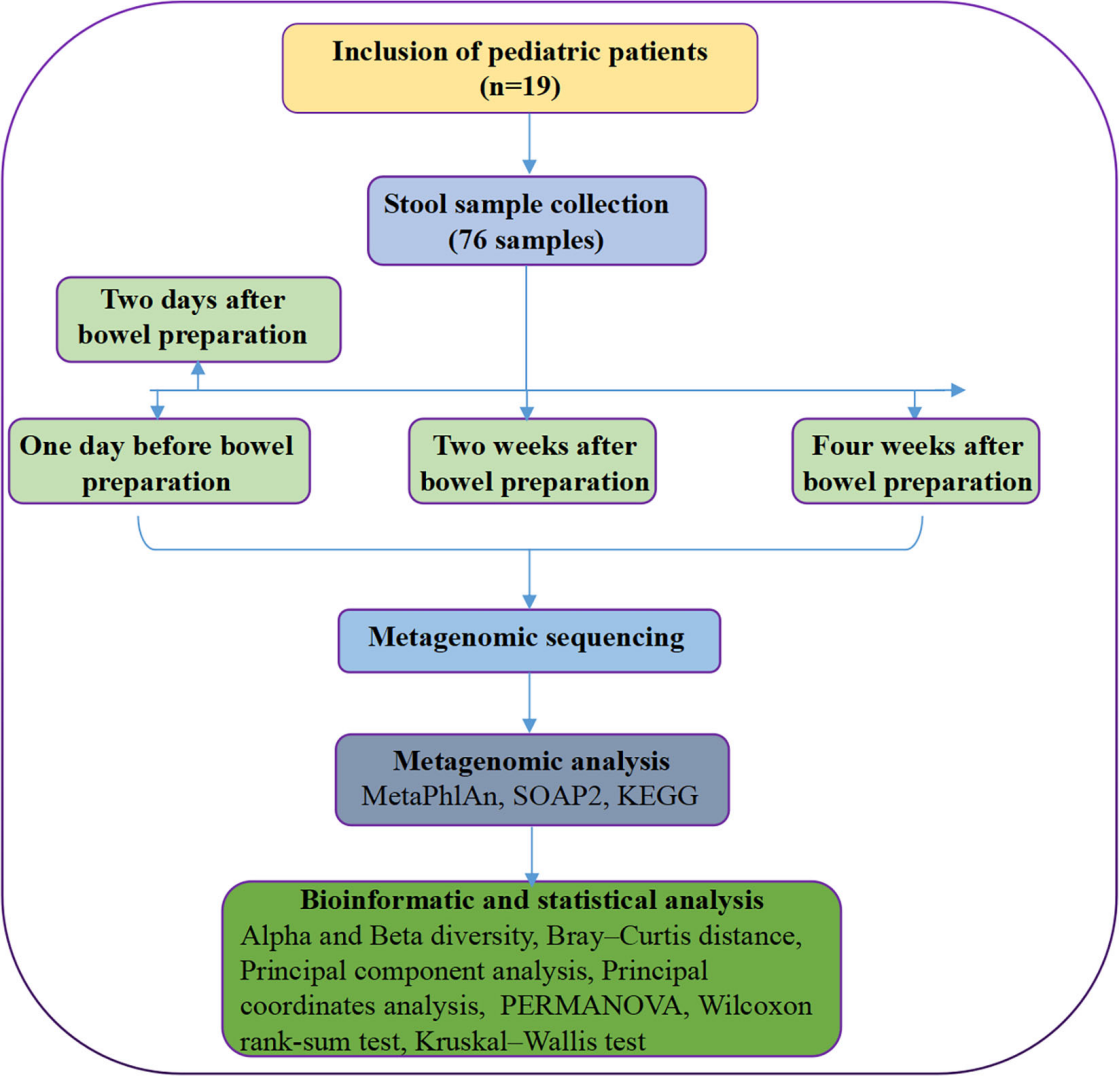
Flowchart of the study design. The samples were collected at 4 time points, including one day before bowel preparation, two days after bowel preparation, two weeks after bowel preparation and four weeks after bowel preparation. Note, KEGG, Kyoto Encyclopedia of Genes and Genomes; PERMANOVA, permutation multivariate analysis of variance.

Fecal samples were obtained from all recruited subjects for metagenomic sequencing. Seventy-six samples of 19 cases were collected in four time periods (Sample ID in **Table S1**). Time_1: one day before bowel preparation; Time_2: two days after bowel preparation (one day after colonoscopy); Time_3: two weeks after bowel preparation; Time_4: four weeks after bowel preparation (**Figure 7**). Each sample was frozen immediately at −80°C before transport to the laboratory within 24 h. Stool sample DNA was extracted using the CTAB method.

A sequencing library was generated using the NEB Next^®^ Ultra™ DNA Library Prep Kit for Illumina (NEB, USA) following the manufacturer’s recommendations, and index codes were added to each sample. The DNA libraries were sequenced on an Illumina platform, and 150 bp paired-end reads were generated.

### 4.3 Metagenomic analysis

The adapter was trimmed, and low-quality reads were filtered using trimmomatic (version 0.39). Then, host sequences were removed by aligning sequencing reads back to the host genome reference (hg38) using soap2 (version 2.20) when sequence identity exceeded 90% (Qin et al., 2012).

Taxonomic profiling of the metagenomic samples was performed using MetaPhlAn (version 3.0.7), which uses clade-specific markers to provide panmicrobial (bacterial, archaeal, viral and eukaryotic) quantification at the species level (Beghini et al., 2021). MetaPhlAn was run with the parameters ‘–read_min_len 50 –add_viruses –unknown_estimation’.

At the same time, the high-quality reads were aligned to the updated gut microbiome gene catalog using SOAP2 (version 2.20) with a threshold of more than 90% identity and 95% read length (Li et al., 2014). The gene abundance profile was calculated as previously described (Li et al., 2014). Next, the relative abundances of KEGG (Kyoto Encyclopedia of Genes and Genomes) orthologous (KO) groups were summed from the relative abundances of their respective genes to obtain a functional profile.

### 4.4 Bioinformatic and statistical analysis

#### 4.4.1 Alpha and beta diversity

Alpha diversity was measured by observed counts and the Shannon index at the gene and species levels with an in-house Perl script. We performed the Wilcoxon rank-sum test for the difference in α diversity. Unless otherwise stated, all statistical analyses were performed in R software, and P values <0.05 were considered statistically significant.

The Bray-Curtis distance was calculated using the Python module scipy (version 1.5.1). Principal component analysis (PCA) was performed using the R package FactoMineR and factoextra. Principal coordinates analysis (PCoA) was used to visualize beta diversity using the Bray-Curtis distance matrix data in R with ggplot2. The R packages vegan and ggplot2 were used to analyse and visualize NMDS (nonmetric multidimensional scaling) using Bray-Curtis distance.

#### 4.4.2 PERMANOVA

PERMANOVA (permutation multivariate analysis of variance) was used to assess the effects of different phenotypes on metagenomic profiles. We used Bray distance and 999 permutations in R (version 3.6.3, vegan package).

#### 4.4.3 Analysis of differential microbiota

We performed a Kruskal-Wallis test for the difference in microbiota. All statistical analyses were performed in R software, and P values < 0.05 were considered statistically significant.

## Data availability statement

The datasets presented in this study can be found in online repositories. The names of the repository/repositories and accession number(s) can be found below: European Bioinformatic Institute (EBI, https://www.ebi.ac.uk/) database under accession code PRJEB56144.

## Ethics statement

The studies involving human participants were reviewed and approved by Ethics Committee of Shenzhen Children’s Hospital. Written informed consent to participate in this study was provided by the participants’ legal guardian/next of kin. Written informed consent was obtained from the individual(s), and minor(s)’ legal guardian/next of kin, for the publication of any potentially identifiable images or data included in this article.

## Author contributions

Conceptualization: YZ, SHZ, SMZ, JZ. Data collection: MC. Formal analysis: SL, QF. Funding acquisition: SMZ, JZ. Project administration: YZ, SHZ, SMZ, JZ. Writing original draft: YZ, SHZ, SMZ, JZ. Writing-review & editing: YZ, SHZ, SMZ, JZ. All authors have read and approved the manuscript. All authors contributed to the article and approved the submitted version.

## Supplementary material

Supplementary Table 1 Sample ID, group and pair of 19 cases.

Supplementary Table 2 Alpha diversity data at the species level.

Supplementary Table 3 The data of differential microbiota at the phylum level.

Supplementary Table 4 The data of differential microbiota at the genus level.

Supplementary Table 5 The data of differential microbiota at the species level.

Supplementary Table 6 KEGG functional pathway data.
